# Screening a Strain of *Aspergillus niger* and Optimization of Fermentation Conditions for Degradation of Aflatoxin B_1_ †

**DOI:** 10.3390/toxins6113157

**Published:** 2014-11-13

**Authors:** Wei Zhang, Beibei Xue, Mengmeng Li, Yang Mu, Zhihui Chen, Jianping Li, Anshan Shan

**Affiliations:** Institute of Animal Nutrition, Northeast Agricultural University, Harbin 150030, China; E-Mails: dianjini2012@163.com (W.Z.); shanshimen@163.com (B.X.); sjzlimengmeng@163.com (M.L.); muyangmyqq@163.com (Y.M.); chenzh1980@163.com (Z.C.)

**Keywords:** *Aspergillus niger*, coumarin, aflatoxin B_1_, degradation

## Abstract

Aflatoxin B_1_, a type of highly toxic mycotoxin produced by some species belonging to the *Aspergillus* genus, such as *Aspergillus flavus* and *Aspergillus parasiticus*, is widely distributed in feed matrices. Here, coumarin was used as the sole carbon source to screen microorganism strains that were isolated from types of feed ingredients. Only one isolate (ND-1) was able to degrade aflatoxin B_1_ after screening. ND-1 isolate, identified as a strain of *Aspergillus niger* using phylogenetic analysis on the basis of 18S rDNA, could remove 26.3% of aflatoxin B_1_ after 48 h of fermentation in nutrient broth (NB). Optimization of fermentation conditions for aflatoxin B_1_ degradation by selected *Aspergillus niger* was also performed. These results showed that 58.2% of aflatoxin B_1_ was degraded after 24 h of culture under the optimal fermentation conditions. The aflatoxin B_1_ degradation activity of *Aspergillus niger* supernatant was significantly stronger than cells and cell extracts. Furthermore, effects of temperature, heat treatment, pH, and metal ions on aflatoxin B_1_ degradation by the supernatant were examined. Results indicated that aflatoxin B_1_ degradation of *Aspergillus niger* is enzymatic and this process occurs in the extracellular environment.

## 1. Introduction

With the growing frequency of food and feed safety issues, there has been an increased focus, specifically, on bio-pollution problems, in which fungal toxins are one of the main factors causing food and feed contamination. Aflatoxins are a group of highly toxic secondary metabolites primarily produced by *Aspergillus flavus* and *Aspergillus parasiticus* [1,2]. Other *Aspergillus* species producing aflatoxin are *A. pseudotamarii* [3], *A. bombycis* [4], and *A. nomius* [5]. Similar to many microbial secondary metabolites, aflatoxins consist of a family of closely related compounds, which include aflatoxin B_1_, B_2_, G_1_, G_2_, M_1_, and M_2_. Aflatoxins, particularly aflatoxin B_1_, demonstrate carcinogenic, teratogenic, hepatotoxic, and immunosuppressive effects on human and animals [6,7].

On the basis of the severe hypertoxicity and wide distribution demonstrated by aflatoxin B_1_, many physical and chemical methods have been applied to inactivate and detoxify this compound in feed systems [8,9]. However, most physical and chemical detoxification methods have their own limitations, such as a loss of the product organoleptic qualities and feed nutritional value, unknown health effects, and the high cost of specific equipment [10]. It has been reported that bacteria and fungi can contribute to the reduction of aflatoxins via biological degradation [11,12]. Moreover, bacteria have been used more often due to advantages such as enhanced degradation within a shorter time period, as well as the production of non-pigments [13]. Several previous reports have described the degradation of aflatoxin B_1_ by many different microorganisms. Farzaneh *et al.* [14] found that *Bacillus subtilis* UTBSP1 could significantly remediate aflatoxin B_1_ in nutrient broth culture and pistachio nuts by 85.66% and 95%, respectively. Many lactic acid bacteria (LAB), such as *Propionibacterium*, *Lactococcus*, *Bifidobacterium*, and *Lactobacillus*, were able to remove aflatoxin B_1_, via a proposed adhesion method [15]. In addition, several other microorganisms, such as *Armillariella tabescens* [16], *Mycobacterium fluoranthenivorans* [17], *Rhodococcus erythropolis* [18], *Stenotrophomonas maltophilia* [19], and *Pseudomonas putida* [20] were reported to degrade aflatoxin B_1_.

The objective of this study was to seek out alatoxin B_1_ degradation microorganisms isolated from microorganism populations of different feed ingredients, to optimize the fermentation conditions and explore the factors affecting degradation efficiency.

## 2. Results and Discussion

### 2.1. Isolation of Microorganisms

Approximately 100 strains were isolated from the vast microbial populations in the feedstuff samples. But only two strains were obtained after screening by using coumarin medium (CM). Coumarin is the basic molecular structure of aflatoxin B_1_. Thus, microorganisms that could utilize coumarin as a carbon source might also be able to use aflatoxin B_1_. The metabolizing processes should result in degradation of the aflatoxin B_1_ [19]. Compared with aflatoxin B_1_, it is much safer for users and cheaper to buy. The coumarin method provided an inexpensive, feasible, and effective tool for selecting aflatoxin B_1_ degradation microorganisms. CM was used in this study for microbial selection. Isolate ND-1 and ND-2 were inoculated onto aflatoxin B_1_-containing nutrient agar (NA) to estimate its ability to remove aflatoxin B_1_. The typical blue fluorescent color disappeared around isolate ND-1 under UV light irradiation after 24 h of incubation. However, for isolate ND-2, the fluorescent color still remained. It indicated that ND-1 could degrade aflatoxin B_1_ and ND-2 could not. This might be due to the ability of ND-2 to utilize glucose in NA as a carbon source rather than aflatoxin B_1_.

### 2.2. Determination of Aflatoxin B_1_ Degradation

**Figure 1:**
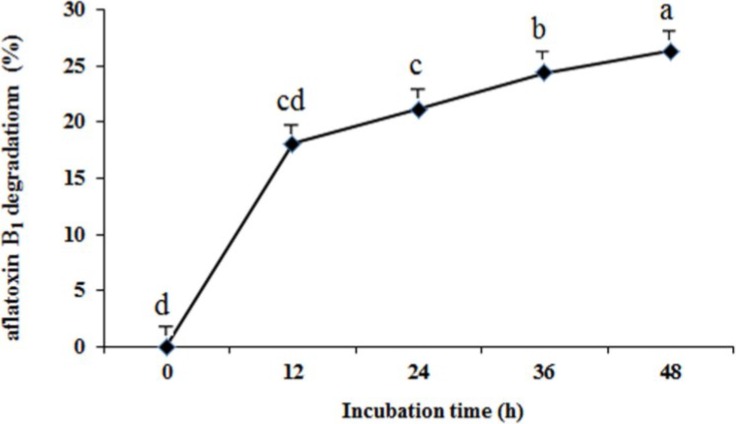
Detrmination of aflatoxin B_1_ degradation by isolate ND-1 after 12, 24, 36, and 48 h incubation. Results are expressed as means ± SEM from three separate experiments. Means without a common letter denote significant differences according Least Significant Difference method (*p <* 0.05); SEM, standard error of the mean. a, b, c, d, indicate significant differences between treatments at the 5% level of probability (*p <* 0.05).

After incubation times of 12, 24, 36, and 48 h, the concentration of aflatoxin B_1_ residue in the culture was detected. The degradation process of aflatoxin B_1_ by ND-1 was relatively slow and continuous, with 18.08% of aflatoxin B_1_ removed in the first 12 h, 21.1% removed after 24 h, 24.4% removed after 36 h, and 26.3% removed after 48 h (Figure 1). Alberts *et al.* reported that a significant (*p <* 0.05) reduction in aflatoxin B_1_ was already observed after 2h in the presence of *Rhodococcus erythropolis* extracellular extracts with only 33.2% residual aflatoxin B_1_ after 72 h [18]. Farzaneh *et al.* found that *Bacillus subtilis* UTBSP1 could significantly remediate aflatoxin B_1_ in nutrient broth culture by 85.66% [14]. Hormisch *et al.* found that the aflatoxin B_1_ concentration was reduced to amounts of 70% to 80% of the initial concentration within 36 h by *Mycobacterium* *fluoranthenivorans* [17]. For ND-1, the percentage of aflatoxin B_1_ degradation was much lower compared to other previously reported strains, which might be due to the difference of strains and the lack of optimization of fermentation conditions.

### 2.3. Identification of Isolate ND-1

**Figure 2:**
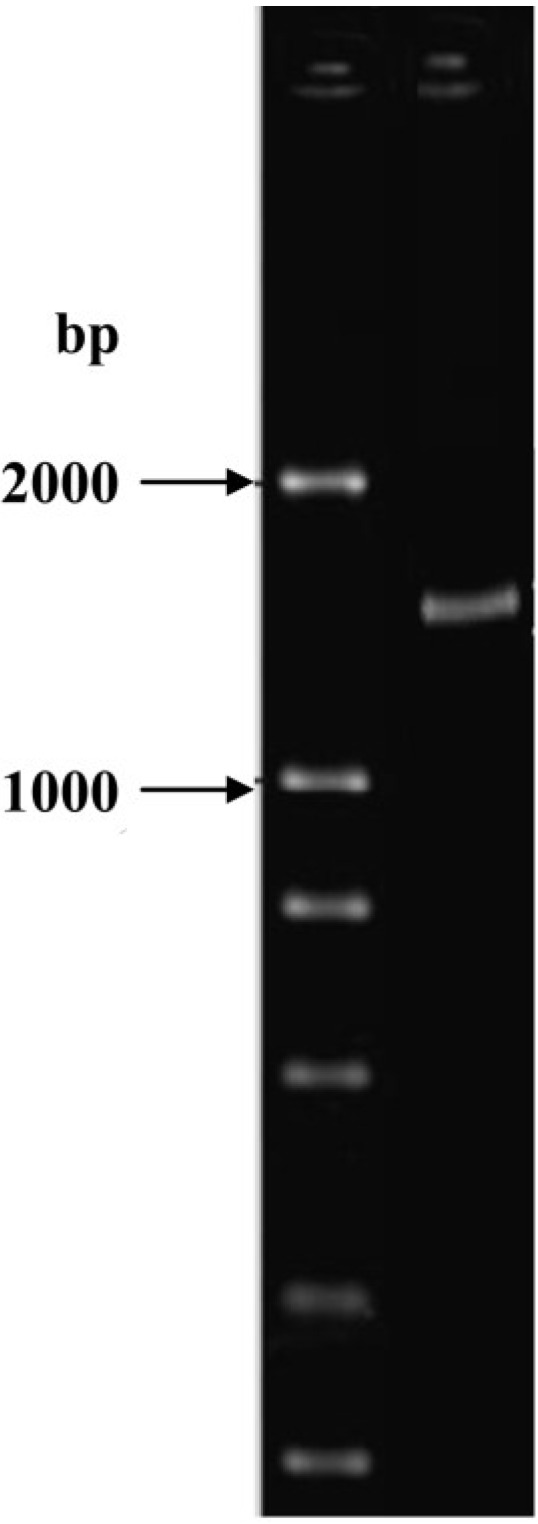
Electrophoresis image of the PCR amplification products of 18S rDNA of isolate ND-1.

**Figure 3:**
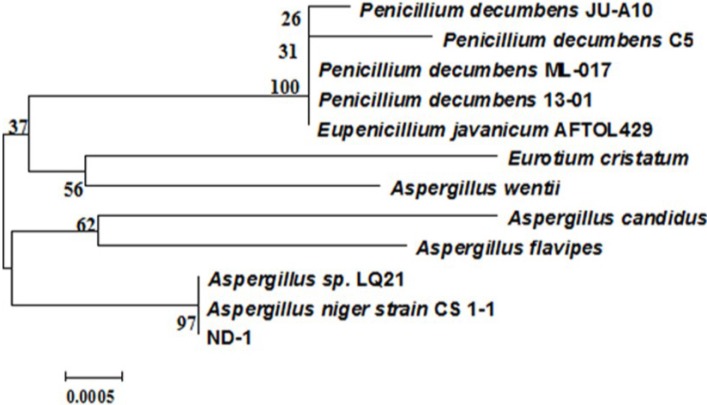
Phylogenetic tree based on the 18S rDNA gene sequences of isolate ND-1 and related taxa.

ND-1 appeared on NA as round fluffy colonies. The conidia color of colonies was black and reverse color of the colonies was yellowish. The conidia shape shown by the strains was globose with smooth conidiophores. Moreover, the conidial head shape was globose and branching pattern was Bi-verticillate. As observed in an electrophoretic image of the PCR amplification product for 18S rDNA of the ND-1 strain, the product was a single band of 1600–1700 bp (Figure 2). The genetic sequence of PCR amplification product was obtained after sequencing. Determination of the 18S rDNA gene sequence using the BLAST Search tool in the GenBankDNA database revealed that the isolate belonged to the genus *Aspergillus* (Figure 3). The closest relationship (99.7% sequence similarity) obtained with the described species was *Aspergillus niger* strain CS 1-1 (GenBank: HM590646.1). This indicated that ND-1 was a strain of *Aspergillus niger*. *Aspergillus niger* has been previously reported to degrade ochratoxin A [21] and zearalenone [22]. However, this is the first report that a fungus in this species exhibits a degradation function in aflatoxin B_1_. This finding provides a new solution for aflatoxin B_1_ degradation. However, optimization of fermentation conditions required further research.

### 2.4. Optimization of Fermentation Conditions for Aflatoxin B_1_ Degradation

When the carbon source was glucose, sucrose, maltose, mannitol, starch, lactose, and galactose, the relevant percentage of aflatoxin B_1_ degradation was 5.7%, 8.5%, 8.0%, 4.9%, 33.5%, 14.9%, and 11.1%, respectively (Table 1). Starch was found to be the most suitable for aflatoxin B_1_ degradation. The degradation was significantly higher than other carbon sucrose. Moreover, it increased with the increased concentration of starch. Among the six different concentrations of starch tested, 4% was the best for aflatoxin B_1_ degradation (Table 1). Different carbon sources affect the growth of microorganisms. The growth of microorganisms was stimulated when the carbon source was utilizable [23]. When the carbon source was difficult to be utilized, the microorganism strains tended to utilize other carbon sources, which might promote a decrease in other carbon-based compounds [24]. Compared to monosaccharides and disaccharides, *Aspergillus niger* had difficulty using starch as a carbon source and selected aflatoxin B_1_ as the carbon source.

**Table 1:** Effect of different carbon sources and starch concentrations on aflatoxin B_1_ degradation (%) by *Aspergillus niger*. The reactions were carried out at 32 °C with agitation at 200 rpm for 24 h. Sterile MTM was used to substitute microbial culture in the control. Results are expressed as means ± SEM of three replicates. Means without a common letter denote significant differences according Least Significant Difference method (*p <* 0.01); SEM, standard error of the mean.

Carbon sources	Aflatoxin B_1_ degradation (%)	Concentrations of starch (%)	Aflatoxin B_1_ degradation (%)
Starch	33.5 ± 1.5 ^A^	4.0	35.0 ± 2.5 ^A^
Lactose	14.9 ± 1.1 ^B^	3.0	34.0 ± 1.5 ^A^
Galactose	11.1 ± 1.6 ^B,C^	2.0	33.1 ± 1.6 ^A^
Sucrose	8.5 ± 0.9 ^B,C^	1.0	19.5 ± 2.2 ^B^
Maltose	8.0 ± 0.7 ^B,C^	0.5	14.0 ± 0.5 ^B,C^
Glucose	5.74 ± 0.4 ^B,C^	0.2	9.0 ± 1.0 ^C^
Mannitol	4.9 ± 0.5 ^C^	-	-

^A, B, C,^ indicate significant differences between treatments at the 1% level of probability (*p <* 0.01).

As shown in Table 2, when the nitrogen source was peptone, proteose peptone, beef extracts, tryptone, beef extract peptone, acidicase peptone, mixed ammonium salt and ammonium nitrate, the relevant percentage of aflatoxin B_1_ degradation was 33.1%, 45.1%, 32.6%, 50.7%, 33.3%, 47.0%, 37.0% and 39.1%, respectively. Tryptone was found to be the most suitable for aflatoxin B_1_ degradation. Among the five different concentrations of tryptone tested, 0.5% was the best for aflatoxin B_1_ degradation. Appropriate nitrogen source and concentration would not only stimulate the growth but also affect the expression of biosynthetic genes and therefore the production of microorganisms [24,25,26].

According to the orthogonal method, the effect of incubation temperature, period and amount of inoculum on aflatoxin B_1_ degradation was analyzed. The order of effects of all factors on aflatoxin B_1_ degradation was temperature > amount of inoculum > time (A > C > B). In terms of the maximum K-value of each column, optimal level of each factor for aflatoxin B_1_ degradation was A2B4C2, corresponding the optimal fermentation conditions included as follows: with the amount of inoculum of 3%, inoculated cultures were incubated at 32 °C for 24 h in a shaker incubator. It has been reported previously that growth and enzyme production of microbes were affected by variations in incubation temperature, period and amount of inoculum [27,28,29].

The initial pH value in MTM showed a significant effect on aflatoxin B_1_ degradation (Table 2). The highest percentage of aflatoxin B_1_ degradation was obtained at pH of 6.0. The pH of medium is a very important environmental factor, which is often neglected. Many investigators claimed that the different morphology of fungi mycelia under a different initial pH value was the critical factor in biomass accumulation and metabolite formation [30,31]. In the present study, the differences among percentage of aflatoxin B_1_ degradation at pH of 6.5–8 were not significant.

**Table 2:** Effect of different nitrogen sources, tryptone concentrations and initial pH value on aflatoxin B_1_ degradation (%) by *Aspergillus niger*. The reactions were carried out at 32 °C with agitation at 200 rpm for 24 h. Sterile MTM was used to substitute microbial culture in the control. Results are expressed as means ± SEM of three replicates. In nitrogen sources group, means without a common letter denote significant differences according Least Significant Difference method (*p <* 0.05); in tryptone concentrations group and initial pH value group, means without a common letter denote significant differences according Least Significant Difference method (*p <* 0.01); SEM, standard error of the mean.

Nitrogen sources	Aflatoxin B_1_ degradation (%)	Concentrations of tryptone (%)	Aflatoxin B_1_ degradation (%)	Initial pH value	Aflatoxin B_1_ degradation (%)
tryptone	50.7 ± 0.6 ^a^	0.5	50.7 ± 0.6 ^A^	6.0	58.2 ± 0.9 ^A^
Acidicase peptone	47.0 ± 1.0 ^b^	0.7	50.4 ± 0.4 ^A^	6.5	50.7 ± 0.6 ^B^
Proteose peptone	45.1 ± 1.5 ^b^	0.9	49.8 ± 0.3 ^A^	7.0	49.2 ± 1.0 ^B^
Ammonium nitrate	39.1 ± 1.1 ^c^	0.3	38.9 ± 1.6 ^B^	7.5	47.9 ± 1.6 ^B^
Mixed ammonium salt	37.0 ± 1.0 ^c^	0.1	14.1 ± 0.9 ^C^	8.0	47.1 ± 1.1 ^B^
Beef extract peptone	33.3 ± 0.8 ^d^	-	-	5.5	35.2 ± 0.9 ^C^
Peptone	33.1 ± 1.6 ^d^	-	-	5.0	27.4 ± 1.8 ^D^
Beef extract	32.6 ± 1.1 ^d^	-	-	-	-

^a, b, c, d,^ indicate significant differences between treatments at the 5% level of probability (*p <* 0.05). ^A, B, C, D,^ indicate significant differences between treatments at the 1% level of probability (*p <* 0.01).

### 2.5. Degradation of Aflatoxin B_1_ by the Supernatant, Cells and Cell Extracts of ND-1

**Figure 4:**
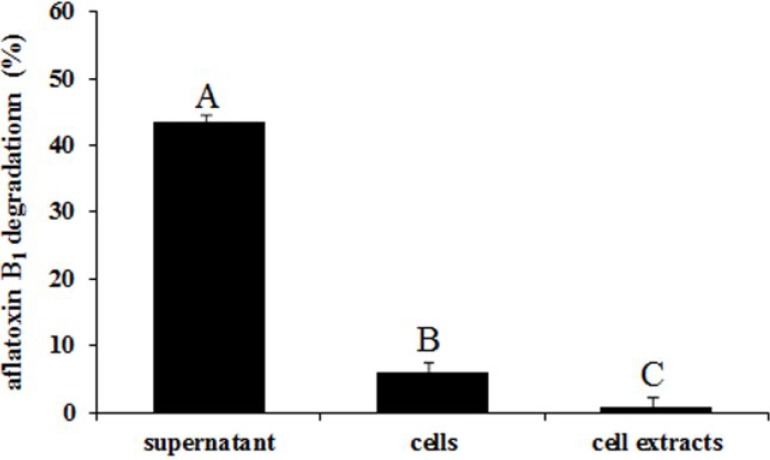
Degradation of aflatoxin B_1_ by the supernatant, cells and cell extracts of *Aspergillus niger* after 24 h incubation. MTM was used to substitute supernatant in the control. The phosphate buffered saline was used to substitute cell and cell extracts in the control. Results are expressed as means ± SEM from three separate experiments. Means without a common letter denote significant differences according Least Significant Difference method (*p <* 0.01); SEM, standard error of the mean. ^A, B, C,^ indicate significant differences between treatments at the 1% level of probability (*p <* 0.01).

The aflatoxin B_1_ degradation activity of ND-1 supernatant was significantly stronger than cells and cell extracts (Figure 4). The supernatant, cells and cell extracts of ND-1 could degrade 43.4%, 5.9%, and 0.8% aflatoxin B_1_, respectively. Some similar results were reported. Culture supernatant of *Stenotrophomonas maltophilia* was able to degrade 78.7% aflatoxin B_1_ after 72 h incubation [19]. The extracellular extracts of *Rhodococcus erythropolis* liquid culture could degrade 66.8% aflatoxin B_1_ after 72 h incubation [18]. In addition, the active ingredient in the culture supernatant was considered to be enzymatic [18].

### 2.6. Effects of Heat Treatment, Temperature, pH, and Metal Ions on Aflatoxin B_1_ Degradation by the Supernatant

**Figure 5:**
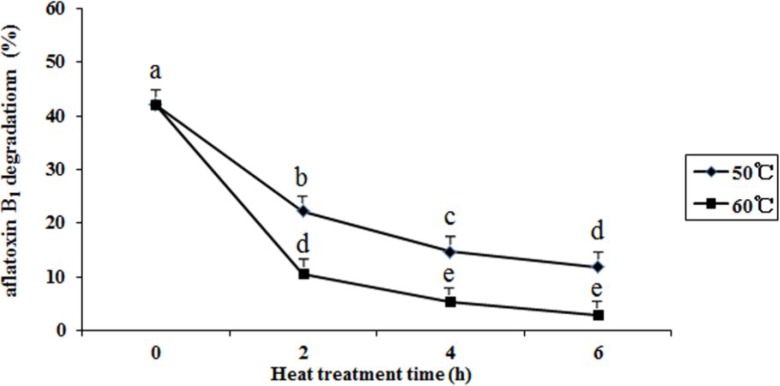
Effect of heat treatment on aflatoxin B_1_ degradation by the supernatant of *Aspergillus niger*. The supernatant was dipped at 50 and 60 °C for 2, 4, and 6 h, respectively. Results are expressed as means ± SEM from three separate experiments. Means without a common letter denote significant differences according Least Significant Difference method (*p <* 0.05); SEM, standard error of the mean. a, b, c, d, e, indicate significant differences between treatments at the 5% level of probability (*p <* 0.05).

The results showed that the percentage of aflatoxin B_1_ degradation decreased from 42.1% to 11.8% and 3.0% when the supernatant dipped at 50 and 60 °C for 6 h, respectively (Figure 5). Heat treatment decreased the aflatoxin B_1_ degradation activities of culture supernatant. The higher the temperature was, the faster the activity decreased. Guan *et al.* reported that when culture supernatant was heated (boiling water bath for 10 min), no degradation activity was observed [19].

**Figure 6:**
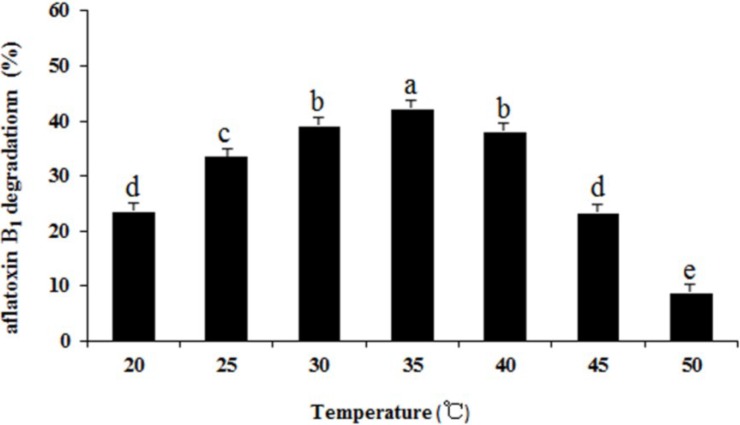
Effect of temperature on aflatoxin B_1_ degradation by the supernatant of *Aspergillus niger*. Results are expressed as means ± SEM from three individual experiments. Means without a common letter denote significant differences according Least Significant Difference method (*p <* 0.05); SEM, standard error of the mean. a, b, c, d, e, indicate significant differences between treatments at the 5% level of probability (*p <* 0.05).

The effect of temperature on aflatoxin B_1_ degradation by the supernatant of ND-1 is shown in Figure 6. The highest percentage of aflatoxin B_1_ degradation (42.1%) was obtained at 35 °C. The aflatoxin B_1_ degradation increased at first but later decreased as the temperature went up from 20 to 50 °C. Temperature effect the growth of microorganisms, thus the metabolism production.

**Figure 7:**
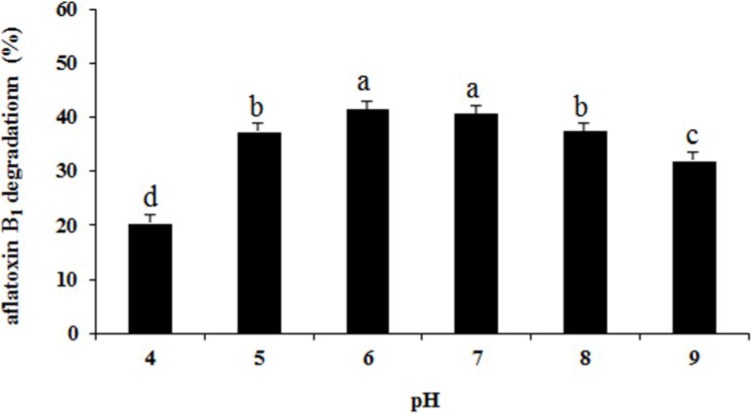
Effect pH on aflatoxin B_1_ degradation by the supernatant of *Aspergillus niger*. Results are expressed as means ± SEM from three separate experiments. Means without a common letter denote significant differences according Least Significant Difference method (*p <* 0.05); SEM, standard error of the mean. a, b, c, d, indicate significant differences between treatments at the 5% level of probability (*p <* 0.05).

The highest percentage of aflatoxin B_1_ degradation (41.4%) was obtained at pH 6.0 and lowest (20.3%) at pH 4.0 (Figure 7). The correlation of aflatoxin B_1_ degradation with pH values is typical for enzymatic reactions. Enzymes have an optimal pH range for maximal activities.

**Figure 8:**
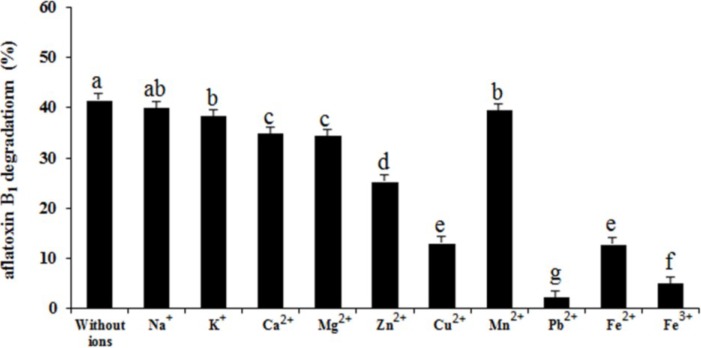
Effect of metal ions on aflatoxin B_1_ degradation by the supernatant of *Aspergillus niger*. The sterilized deionized water was added to the reaction mixture in treatment without ions. Results are expressed as means ± SEM from three individual experiments. Means without a common letter denote significant differences according Least Significant Difference method (*p <* 0.05); SEM, standard error of the mean. a, b, c, d, e, f, g, indicate significant differences between treatments at the 5% level of probability (*p <* 0.05).

The effect of different metal ions on aflatoxin B_1_ degradation is shown in Figure 8. Compared to the treatment without ions (41.4%), all metal ions decreased the percentage of aflatoxin B_1_ degradation. Ions Pb^2+^ showed the strongest inhibition function (*p <* 0.001). Similar results were reported that Cu^2+^, Mn^2+^ and Zn^2+^ inhibited aflatoxin B_1_ degradation by *Flavobacterium aurantiacum* [32]. But contrary to the results of our study, D’Souza and Brackett (2000) reported that incubating cells with 0.1, 1 and 10 mM Ca^2+^ for 48 h significantly increased aflatoxin B_1_ degradation by 11.8%, 13.5%, and 14.0%, respectively [33]. Likewise, incubation with 0.1, 1, and 10 mM Mg^2+^ for 48 h significantly increased aflatoxin B_1_ degradation by 13.8%, 13.3%, and 13.1%, respectively. The effects of metal ions on degradation activity further supported the enzyme involvement in aflatoxin B_1_ degradation by the isolate.

## 3. Materials and Methods

### 3.1. Culture Media

Each liter of coumarin medium (CM) contained 1.0 g coumarin (Aladdin, Shanghai, China), 2.0 g (NH_4_)_2_SO_4_, 0.2 g MgSO_4_·7H_2_O, 1.5 g Na_2_HPO_4_·12H_2_O, 0.001 g FeSO_4_·7H_2_O, 0.01 g CaCl_2_·2H_2_O and 1.5 g KH_2_PO_4_. The pH of the medium was adjusted to 7.2. NB consisted of 3.0 g yeast extract, 5.0 g peptone, 6.0 g glucose, 10.0 g NaCl per liter (pH 7.0). NA, which was NB plus 15 g agar, was used for preserving microbial isolates. Each liter of modified Thayer-Martin medium (MTM) contained 5.0 g peptone, 1.0 g K_2_HPO_4_, 2.0 g yeast extract, 0.5 g MgSO_4_ and 20.0 g glucose. The pH of the medium was adjusted to 6.4.

### 3.2. Isolation of Microorganisms

One hundred and sixteen samples were selected for aflatoxin B_1_ degradation. The samples consisted of 16 corn, 12 distillers dried grains with solubles, 10 corn gluten meal, eight corn germ meal, 12 maize gluten feed, 16 wheat bran, 13 wheat germ meal, 14 soybean meal, and 15 types of meal collected from eight regions of Northeastern China where the feed ingredients might be affected by mycotoxin contamination.

Each pulverized sample (2.0 g) were dissolved in normal saline (10 mL), and then fully vortexed for 1 min. Supernatant (0.1 mL) was transferred to NB (10 mL) medium, and cultured for 24 h at 37 °C. The microbe liquid (0.2 mL) was plated on plates of NA, which was incubated at 37 °C until visible colonies appeared. Single colonies were isolated and transferred to fresh NA for six times. Colonies were preserved as pure isolates on NA.

Screening microbes for aflatoxin B_1_ degradation was carried out in CM. After 72 h of culture at 32 °C, strains that could grow in CM were selected for rescreening. This procedure was repeated three times.

Rescreening microbes for aflatoxin B_1_ degradation was carried out following the K-B disk diffusion method of CLSI-approved standard M02-A10 [34], with slight modifications. 0.01g aflatoxin B_1_ standard (Sigma, St. Louis, MO, USA) was dissolved in sterilized deionized water (1000 mL) to a stock solution of 10,000 ppb. Sterilized aflatoxin B_1_ solution (0.4 mL) was coated uniformly on NA. A 6-mm diameter sterilized filter paper dipped in each strains culture broth was carefully placed on top of the NA. After 24 h of incubation at 37 °C, the plates of NA were exposed to UV light irradiation to observe the change of fluorescent color. Strains that the fluorescence around them disappeared were selected and tested for aflatoxin B_1_ degradation.

### 3.3. Determination of Aflatoxin B_1_ Degradation

The degradation of aflatoxin B_1_ by the selected strains was carried out in NB. Twelve-hour pre-inoculum (2.4 mL) was inoculated in NB (77.6 mL) containing aflatoxin B_1_ (10 ppb). Inoculated cultures were incubated at 32 °C with agitation at 200 rpm for 0, 12, 24, 36, and 48 h in a shaker incubator (Harbin Donglian Electronic Equipment Inc., Harbin, China). After incubation, the cells were removed by centrifugation at 10,000 g for 5 min (Allegra 64R, Beckman Coulter Inc., Brea, CA, USA). Sterile NB was used to substitute microbial culture in the control. The resulting supernatant was used for aflatoxin B_1_ analysis. Sterile NB was used to substitute microbial culture in the control.

The concentration of aflatoxin B_1_ in the culture medium was determined with an aflatoxin B_1_ test kit using the ELISA method (aflatoxin B_1_ FTRT, ELISA Technologies, Inc., Gainesville, FL, USA). The reaction mixtures were extracted with methanol (80%) by mixing gently for 10 min at room temperature. The methanol extracts were evaporated under a mild stream of nitrogen at 50 °C, the residue was dissolved in 2.0 mL of sample dilution buffer. 50 μL aflatoxin B_1_ standard solutions and 50 μL prepared test samples were added into separate wells of microtiter plate, in duplicate. Then, 25 μL of conjugate (Aflatoxin-HRP) and 25 μL of antibody solution were added to each well, mixed gently and incubated for 1 h in the dark at 37 °C. The liquid was then removed completely from the wells; the each well was washed with 250 μL rinsing buffer. The washing procedure was repeated for three times. After the washing step, 100 μL TMB substrate solution was added to each well and incubated for 30 min at room temperature (20–25 °C). Finally, 100 μL of the stop solution was added to each well and the absorbance was measured at 450 nm in ELISA reader (TECAN Inc., Durham, NC, USA). The percentage of aflatoxin B_1_ degradation (D) was calculated using the following formula:

*D* = [(*X_3_* − *X_2_* ) − ( *X_3_* − *X_1_*)] *X_3_*^−1^ × 100% = (*X_1_* − *X_2_*) *X_3_*^−1^ × 100% (1)

where:

*X_1_*: The concentration of aflatoxin B_1_ in the control after fermentation;
*X_2_*: The concentration of aflatoxin B_1_ in treatment after fermentation;
*X_3_*: The initial concentration of aflatoxin B_1_ before fermentation.

### 3.4. Identification of the Aflatoxin B_1_ Degradation Strains

The isolates were cultured for 7 days on NA at 37 °C. Pigment production and the colony characteristics were noted. The conidia, hyphae, conidial head, conidiophores, spores, *etc.* were observed microscopically for morphological identification. DNA was extracted by using the GenEluteTM Kit (Bacterial Genomic DNA Kit, Sigma, St. Louis, MO, USA) according to the manufacturer’s instructions. 18S rDNA, was amplified by PCR using universal primers (5'-GTAGTCATATGCTTGTCTC-3'; 5'-TCCGCAGGTTCACCTACGGA-3') [35]. The amplified product was analyzed using 1.2% agarose gel electrophoresis. Purified DNA was sequenced by Invitrogen (Shanghai, China). The sequences were aligned with those in GenBank using the Blast program to determine the closest known relatives of the partial 18S rDNA gene sequences obtained. The generated DNA sequences and sequences derived from GenBank were aligned using the ClustalX program [36]. Neighbor joining analysis and calculation of bootstrap values were performed using the MEGA program.

### 3.5. Optimization of Fermentation Conditions

All optimization experiments were conducted in MTM. 12 h pre-inoculum (2.4 mL) was inoculated in MTM (77.6 mL) containing aflatoxin B_1_ (10 ppb). Inoculated cultures were incubated at 32 °C with agitation at 200 rpm for 24 h in a shaker incubator. After incubation, the cells were removed by centrifugation at 10,000 *g* for 5 min. The resulting supernatant was used for aflatoxin B_1_ analysis. Sterile MTM was used to substitute microbial culture in the control. The aflatoxin B_1_ degradation tests were performed as described in Section 3.3.

#### 3.5.1. Optimization of Fermentation Medium

For the carbon source tests, peptone was added as the nitrogen source during the tests. 20% glucose, sucrose, maltose, mannitol, starch, lactose, and galactose were individually introduced into MTM to determine the effect of the carbon source on aflatoxin B_1_ degradation.

The effect of different concentrations of the best carbon source on aflatoxin B_1_ degradation was studied in MTM with 0.2%, 0.5%, 1.0%, 2.0%, 3.0%, and 4.0% of optimized carbon source.

For the nitrogen source tests, the optimized carbon source was added as the carbon source instead of glucose. 0.5% peptone, proteose peptone, beef extract, tryptone, beef extract peptone, acidicase peptone, mixed ammonium salt (NH_4_NO_3_:(NH_4_)_2_SO_4_ = 1:1), and ammonium nitrate were individually introduced into the MD medium to determine the effect of the nitrogen source on aflatoxin B_1_ degradation.

The effect of different concentrations of the best nitrogen source on aflatoxin B_1_ degradation was studied in MTM with 0.1%, 0.3%, 0.5%, 0.7%, and 0.9% of optimized nitrogen source.

#### 3.5.2. Optimization of Incubation Temperature, Period, Amount of Inoculum and pH

The orthogonal L_16_ (4^3^) was used to optimize incubation temperature, period and amount of inoculum. The levels of each factor are listed in Table 3.

In the pH tests, initial pH value in MTM was adjusted to 5.0, 5.5 and 6.0 by using HCl, and to 6.5, 7.0, 7.5, and 8.0 by using NaOH.

**Table 3:** Orthogonal optimization of different incubation temperature, period and amount of inoculum for the aflatoxin B_1_ degradation by ND-1. Sterile MTM was used to substitute microbial culture in the control.

Factors	A (°C)	B (h)	C (%)
Level 1	28	12	1
Level 2	32	24	3
Level 3	36	36	5
Level 4	40	48	7

Symbols A, B and C represent factors of incubation temperature, period and amount of inoculum.

### 3.6. Degradation of Aflatoxin B_1_ by the Supernatant, Cells and Cell Extracts of Strains

Twelve-hour pre-inoculum (2.4 mL) was inoculated in MTM (77.6 mL) in a 100 mL flask. Inoculated cultures were incubated at 32 °C with agitation at 200 rpm for 24 h in a shaker incubator. The preparation of supernatant, cell and cell extracts was carried out following the method of Teniola *et al.* [37]. The supernatant and cell were obtained by the following procedures. Cells were pelleted by centrifugation at 10,000 *g* for 5 min. Cell pellets were washed twice with phosphate buffered saline (pH 7.4). The cell extracts were harvested by the following procedures. The cell pellets were resuspended in phosphate buffered saline (pH 7.4) in preparation for cell rupture. The suspension was disintegrated thrice by using an ultrasonic cell disintegrator (Shanghai Xinzhi Instruments Inc., Shanghai, China). The cell disruption steps were carried out on ice. The disintegrated cell suspension was centrifuged at 12,000 g for 20 min at 4 °C. Supernatant from the centrifugation step was filtered aseptically using sterile cellulose pyrogen free disposable filters of 0.2 μm pore size. Aflatoxin B_1_ stock solution was added to each reaction liquid to reach a final concentration of 10 ppb. The degradation tests were carried out after incubation at 32 °C with agitation at 200 rpm for 24 h in a shaker incubator as described in 3.3. MTM was used to substitute supernatant in the control. The phosphate buffered saline was used to substitute cell and cell extracts in the control.

### 3.7. Effects of Heat Treatment, Temperature, pH and Metal Ions on Aflatoxin B_1_ Degradation by the Supernatant

The aflatoxin B_1_ degradation tests by the supernatant were performed as described in 3.6. MTM was used to substitute supernatant under the following conditions in the control.

The effect of heat treatment was determined by dipping the supernatant at 50 and 60 °C for 2, 4, and 6 h, respectively.

To measure the effect of temperatures, the reaction mixture was incubated at 20, 25, 30, 35, 40, 45, and 50 °C with agitation at 200 rpm for 24 h in a shaker incubator.

In the pH tests, initial pH value was adjusted to 4.0, 5.0 and 6.0 by using HCl, and to 7.0, 8.0, and 9.0 by using NaOH. The reaction mixture was incubated at 32 °C with agitation at 200 rpm for 24 h in a shaker incubator.

To evaluate the effect of different metal ions, Li^+^, Na^+^, K^+^, Ca^2+^, Mg^2+^, Zn^2+^, Cu^2+^, Mn^2+^, Pb^2+^, Fe^2+^ and Fe^3+^ (in the form of LiCl, NaCl, KCl, CaCl_2_, MgSO_4_, ZnCl_2_, CuSO_4_, MnCl_2_, Pb(NO_3_)_2_, FeSO_4_, and FeCl_3_) was adding to the reaction mixture respectively in a final concentration of 5 mM. The sterilized deionized water was added to the reaction mixture in treatment without ions. The reaction mixture was incubated at 32 °C with agitation at 200 rpm for 24 h in a shaker incubator.

### 3.8. Statistical Analysis

All specific experiments were repeated three times. Statistical analysis was performed using Statistix 8.1 (Analytical Software, Tallahassee, FL, USA, 2005). Analysis of variance (ANOVA) was performed to detected significance. Significant differences between means were determined using the Least Significant Difference method.

## 4. Conclusions

We employed a method using coumarin as a selective agent to search for aflatoxin B_1_ degradation microorganisms in this study. A strain of *Aspergillus niger* that could degrade 26.3% of aflatoxin B_1_ after 48 h of fermentation in NB was obtained. The degradation rose to 58.2% under the optimal fermentation conditions. The aflatoxin B_1_ degradation activity of *Aspergillus niger* culture supernatant was significantly stronger than cells and cell extracts and affected by heat treatment, temperature, pH, and metal ions. The results indicated that the aflatoxin B_1_ degradation of *Aspergillus niger* is enzymatic and this process mainly occurs in the extracellular environment.

## Supplementary Materials

Supplementary materials can be accessed at: http://www.mdpi.com/2072-6651/6/11/3157/s1.

## Supplementary Files

Supplementary File 1
