# Supplementary material for: Screening a Strain of Aspergillus niger and Optimization of Fermentation Conditions for Degradation of Aflatoxin B1
Source: Toxins (Basel). 2014 Nov 13;6(11):3157–72. doi: 10.3390/toxins6113157 (PMC4247255; doi:10.3390/toxins6113157)

Supplementary Information

Table S1. Results and analysis of L_{16} (4^3) orthogonal test of aflatoxin B_1 degradation by ND-1.

Run	A	B	C	Aflatoxin B_1 Degradation (%)
1	1	1	1	18.0
2	2	2	1	24.5
3	3	3	1	33.3
4	4	4	1	22.4
5	1	2	2	38.2
6	2	1	2	38.9
7	3	4	2	38.1
8	4	3	2	21.0
9	1	3	3	26.4
10	2	4	3	48.6
11	3	1	3	22.0
12	4	2	3	14.3
13	1	4	4	29.0
14	2	3	4	29.6
15	3	2	4	26.6
16	4	1	4	12.3
K1	111.5	101.2	98.3	-
K2	141.5	103.6	126.1	-
K3	120.0	110.3	121.3	-
K4	70.1	128.0	97.5	-
k1	27.9	25.3	24.6	-
k2	35.4	25.9	31.5	-
k3	30.0	27.6	30.3	-
k4	17.5	32.0	24.4	-
r	17.9	6.7	7.2	-

Symbols A, B and C represent factors of incubation temperature, period and amount of inoculum. Symbols 1, 2, 3 and 4 represent different levels of incubation temperature, period and amount of inoculum.

Figure S1. Degradation of aflatoxin B_1 by ND-1 in plate assay.

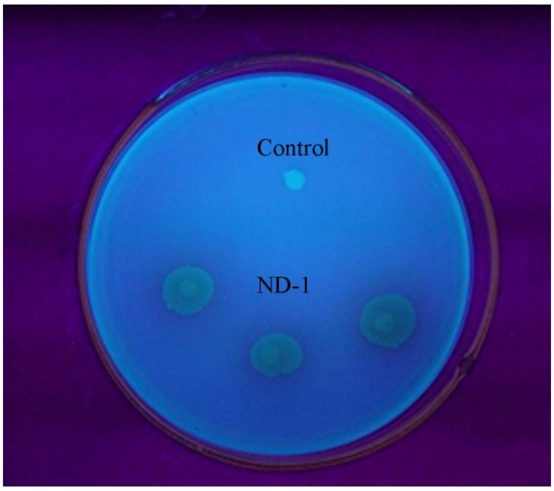

Figure S2. Degradation of aflatoxin B_1 by ND-2 in plate assay.

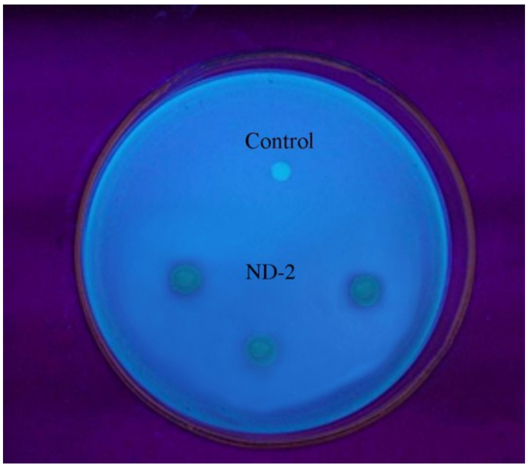

Supplement: Supplementary File 1 [file toxins-06-03157-s001.pdf]
